# The lectin-like domain of TNF reduces pneumonia-induced injury in the perfused human lung

**DOI:** 10.1172/jci.insight.188325

**Published:** 2025-06-09

**Authors:** Mazharul Maishan, Hiroki Taenaka, Bruno Evrard, Shotaro Matsumoto, Angelika Ringor, Carolyn Leroux, Rudolf Lucas, Michael A. Matthay

**Affiliations:** 1 Cardiovascular Research Institute and; 2 Division of Pulmonary, Critical Care, Allergy and Sleep Medicine, Department of Medicine, UCSF, San Francisco, California, USA.; 3 Inserm CIC 1435, Dupuytren Teaching Hospital, Limoges, France.; 4 Vascular Biology Center,; 5 Department of Pharmacology and Toxicology, and; 6 Division of Pulmonary Critical Care and Sleep Medicine, Medical College of Georgia, Augusta University, Augusta, Georgia, USA.; 7 Department of Medicine and; 8 Department of Anesthesiology, UCSF, San Francisco, California, USA.

**Keywords:** Infectious disease, Pulmonology, Therapeutics, Bacterial infections, Sodium channels

## Abstract

Bacterial pneumonia is the most common cause of acute respiratory distress syndrome (ARDS), characterized by disrupted pulmonary endothelial barrier function, hyperinflammation, and impaired alveolar epithelial fluid clearance. ARDS has a high mortality rate and no proven pharmacological treatments, stressing the need for new targeted therapies. The TIP peptide, mimicking the lectin-like domain of TNF, directly binds to the α subunit of the epithelial Na^+^ channel, expressed in both alveolar epithelial and capillary endothelial cells, and may increase lung endothelial barrier function and alveolar fluid clearance during bacterial infection. This study tested these potential therapeutic mechanisms of the TIP peptide in a clinically relevant preparation of the ex vivo–perfused human lung injured by *Streptococcus pneumoniae*. Therapeutic administration of the TIP peptide reduced pulmonary barrier permeability to protein and lung edema formation, increased alveolar edema fluid clearance, and produced an antiinflammatory effect in the airspaces with reductions in IL-6 and IL-8 levels. Additionally, the TIP peptide reduced the translocation of bacteria into the circulation. These findings establish 3 mechanisms of benefit with the TIP peptide to reduce injury in the human lung and support the clinical relevance as a potential therapeutic for pneumococcal bacterial pneumonia.

## Abstract 2

The TIP peptide is a potential therapeutic that reduced lung injury and edema formation in donor human lungs that were injured by Streptococcus pneumoniae.

## Introduction

Acute respiratory distress syndrome (ARDS) has a substantial health burden and high mortality rate, with lung-protective mechanical ventilation and prone positioning being the only proven interventions, as no pharmacological cure exists (1). Bacterial pneumonia is a major risk factor for developing ARDS, and Gram-positive *Streptococcus pneumoniae* (*S*. *pneumoniae*) is the primary causative pathogen in many patients (2). Additionally, ARDS treatment is complicated by the multifactorial pathophysiological mechanisms that lead to lung injury and respiratory failure (3). Several clinical trials of various pharmacological agents targeting different disease mechanisms have been conducted, but no effective therapy has emerged to date (4). One of the targeted mechanisms is the formation of edema fluid in the distal airspaces of the lung, resulting from pulmonary barrier disruption, which enables proteinaceous fluid to extravasate from the circulation into the airspaces, and a concomitant decrease in the rate of alveolar fluid clearance (AFC) that would normally remove this fluid (5). Edema in the airspaces disrupts gas exchange, and impaired AFC correlates with mortality in patients with ARDS (6).

The alveolar epithelium is a very tight barrier that restricts fluid movement and actively facilitates fluid clearance from the airspaces primarily through vectorial Na^+^ transport by alveolar type II cells via the apical epithelial Na^+^ channel (ENaC) and basolateral Na^+^/K^+^-ATPase (7). The resulting Na^+^ gradient drives the movement of water across the alveolar epithelium from the airspaces into the interstitium, through paracellular pathways and transcellular aquaporin channels on alveolar type I cells (8). ENaC in its native form possesses 3 subunits (α, β, and γ) (9), of which the α subunit is the most important for AFC to keep the airspaces dry (10). Thus, targeting ENaC function may be a promising direction for treatment of ARDS, by enhancing the clearance of edema fluid from the airspaces, strengthening alveolar-capillary barrier function, and reducing edema formation (11).

Apart from live bacteria, bacterial toxins such as the pneumococcal pore-forming cytolysin pneumolysin (PLY) and LPS generated by Gram-negative pathogens directly contribute to the loss of AFC (12, 13). Specifically, PLY reduces the open probability of ENaC and decreases the surface expression of ENaC on the alveolar epithelium (14, 15). Moreover, inflammatory cytokines, including TNF, can impair ENaC function because TNF receptor 1 activation causes transcriptional inhibition of the 3 ENaC subunits and posttranslational inhibition of ENaC-α (16). However, TNF can also increase Na^+^ uptake in alveolar epithelial cells in vitro (17) and increase AFC in different models of pulmonary edema (18–21). Although opposite effects of the 2 TNF receptor types can be involved (22), the dichotomous functions of TNF can also be explained by distinct functional domains within the TNF protein, the TNF receptor 1 binding site on the one hand and the lectin-like domain on the other hand (23, 24). A 17–amino acid sequence, termed the TIP peptide (also known as AP-301 and solnatide), mimics this lectin-like domain of TNF and directly binds to ENaC-α to increase the open probability (14) and current flow through the channel (15). Accordingly, the TIP peptide increases Na^+^ uptake in alveolar type II cells (25, 26), enhances AFC, and reduces lung injury in several animal models (23, 27–29).

Given the potential value of targeting ENaC function to enhance AFC for treating ARDS and the promising results of the TIP peptide studied in preclinical animal models, this study was designed to test the TIP peptide in a translational model of ARDS. In these experiments, the ex vivo–perfused human lung preparation (30) was used to test the effect of the TIP peptide in a model of bacterial pneumonia with live *S*. *pneumoniae* that was recently developed within our group (31). This ex vivo perfusion preparation has been used extensively to study mechanisms of injury in the human lung and to test potential therapeutics for ARDS (32–34).

## Results

### S. pneumoniae instilled into the distal airspaces causes injury in the ex vivo–perfused human lung.

**Figure 1:**
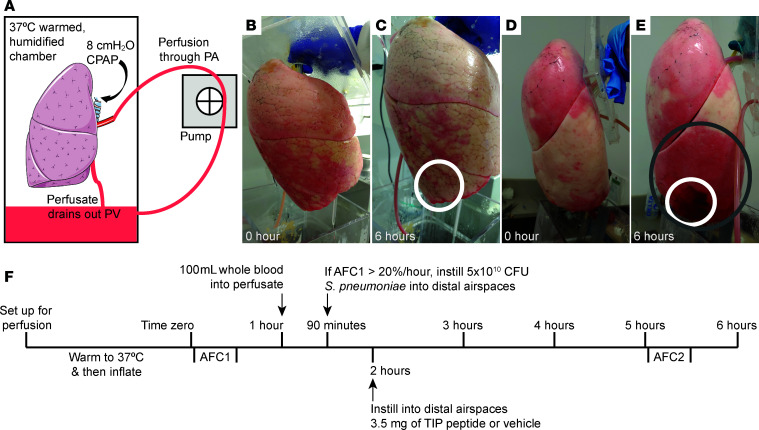
A translational model of bacterial pneumonia using the ex vivo–perfused human lung injured by *S*. *pneumoniae*. (**A**) Schematic showing the human lung perfusion preparation. Either the right or the left lung from a donor not accepted for transplant was selected for perfusion based on the absence of injury by gross appearance. A perfusate mixture of physiological medium and fresh whole blood was circulated by a roller pump through the cannulated pulmonary artery (PA) and drained freely out of the pulmonary vein (PV). Once warmed to 37°C, the lung was inflated with 8 cmH_2_O of continuous positive airway pressure (CPAP) through an endobronchial tube secured to the main bronchus. (**B**–**E**) Photographs of representative human lungs that were perfused ex vivo and inflated with CPAP. The 2 photographs on the left show a human lung, immediately after inflation at the beginning of the experiment (**B**) and after 6 hours of perfusion (**C**), from the control group, which did not receive bacteria but only saline vehicle (white circle). The 2 photographs on the right show a human lung from a different donor immediately after inflating (**D**), instilled with 5 × 10^10^ colony-forming units (CFU) of *S*. *pneumoniae* after 1.5 hours, and perfused for a total of 6 hours (**E**), resulting in visible infiltrates in the region where bacteria were instilled (white circle) and spreading of injury through the lower lobe (gray circle). (**F**) Experimental timeline for ex vivo human lung perfusion studies with measurements of AFC in a separate set of experiments to test the effect of the TIP peptide targeting the ENaC. After inflating the lung, AFC was measured, and the perfusion experiment was continued if this initial AFC was greater than 20% per hour to ensure the donor lung had relatively normal AFC function at baseline.

Either the left or the right lung from a donor not accepted for transplant was housed in a warmed and humidified chamber, and 37°C perfusate was circulated by a roller pump through the cannulated pulmonary artery and freely drained out of the pulmonary vein (Figure 1A). Once the lung was inflated, 100 mL of fresh whole blood was added to the perfusate, and then 5 × 10^10^ colony-forming units (CFU) of *S*. *pneumoniae* suspended in saline were instilled into the distal airspaces of the lower lobe. After 4.5 hours, there was evidence of injury in the localized region where the bacteria were instilled and spreading of injury throughout the lower lobe (Figure 1, D and E) as compared with control lungs in which only saline vehicle was instilled but not bacteria (Figure 1, B and C). In a separate series of experiments, AFC was measured at 2 points during perfusion (Figure 1F), prior to and 3.5 hours after instillation of bacteria or vehicle, to quantify the change in alveolar epithelial function over time.

### S. pneumoniae causes edema formation in human lungs, which is reduced by the TIP peptide.

**Figure 2:**
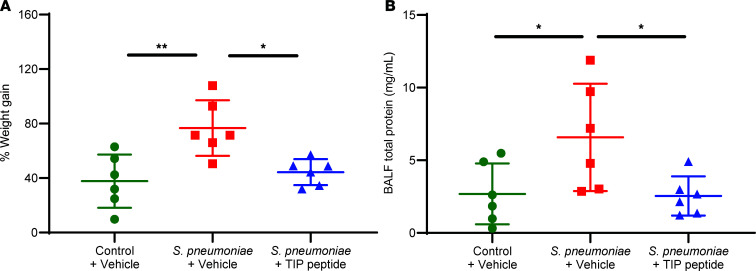
Treatment with the TIP peptide reduces pulmonary edema and barrier dysfunction induced by *S*. *pneumoniae*. (**A**) In a separate set of perfusion studies, AFC was not measured, and instead the weight of the lung prior to and after 6 hours of perfusion was measured to calculate a percentage weight gain during perfusion, which was used as an index of pulmonary edema formation. The TIP peptide instilled into the distal airspaces significantly reduced the weight gain induced by *S*. *pneumoniae*, to levels comparable to those in control lungs. (**B**) Bronchoalveolar lavage (BAL) was performed after 6 hours of perfusion to measure the total protein concentration in the cell-free BAL fluid (BALF), an index of pulmonary barrier integrity. *S*. *pneumoniae* instilled into the distal airspaces significantly increased total protein concentration in the BALF, indicating disruption of pulmonary barrier function. The TIP peptide reversed this disruptive effect of the bacteria, as BALF total protein concentrations were significantly reduced to levels comparable to those of control lungs. *P < 0.05, **P < 0.01, 1-way ANOVA with Tukey’s multiple comparisons test for replicate experiments, with *n* = 6 for each group shown in **A** and **B**.

In a batch of donor lungs in which AFC was not measured, each lung was weighed prior to and after 6 hours of perfusion to calculate the percentage weight gain (Figure 2A), used as a surrogate measure of pulmonary edema formation during perfusion. *S*. *pneumoniae* instilled into the distal airspaces caused significantly greater weight gain than in control lungs that did not receive bacteria (Figure 2A). At 30 minutes following the instillation of *S*. *pneumoniae*, 3.5 mg of the TIP peptide suspended in saline was administered into the same region of the distal airspaces in the lower lobe where *S*. *pneumoniae* were instilled. The TIP peptide treatment significantly reduced the bacteria-induced weight gain to levels comparable to the weight gain measured in control lungs that did not receive bacteria.

### The TIP peptide reduces S. pneumoniae–induced pulmonary barrier permeability to protein.

Following the measurement of lung weight after 6 hours of perfusion, bronchoalveolar lavage (BAL) sampled the region of the distal airspaces where *S*. *pneumoniae* or the vehicle was instilled. Compared with control lungs that did not receive bacteria, lungs instilled with *S*. *pneumoniae* had a significantly greater concentration of total protein in the cell-free BAL fluid (BALF) compared with that of control lungs (Figure 2B), indicating an increase in pulmonary barrier protein permeability caused by the bacteria. The TIP peptide, administered 30 minutes after *S*. *pneumoniae* instillation, significantly reduced the total protein concentration in the BALF compared with injured lungs that did not receive the TIP peptide (Figure 2B).

### The TIP peptide rescues AFC disrupted by S. pneumoniae in the human lung.

**Figure 3:**
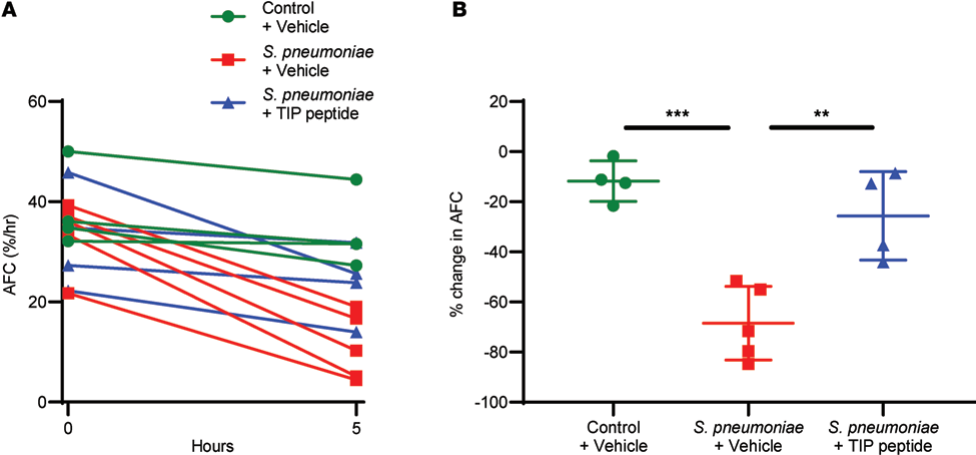
The TIP peptide rescues AFC in the human lung injured by *S*. *pneumoniae*. (**A**) The AFC that was measured for each ex vivo–perfused human lung at baseline (0 hour) and after 5 hours of perfusion. At 1.5 hours, either 5 × 10^10^ CFU of *S*. *pneumoniae* or the saline vehicle only (control group) was instilled into the lower lobe of the human lung. At 2 hours, either 3.5 mg of TIP peptide or the saline vehicle only was instilled into the same region of the lower lobe where bacteria were instilled. (**B**) The calculated percentage change in AFC from baseline (0 hour) to 5 hours of perfusion for each lung presented in **A**. Human lungs that were instilled with *S*. *pneumoniae* had a significantly greater decrease in AFC during perfusion than control lungs that did not receive bacteria. Instillation of the TIP peptide significantly reduced this decrease in AFC caused by *S*. *pneumoniae*, to levels comparable to those of control lungs. **P < 0.01, ***P < 0.001, 1-way ANOVA with Tukey’s multiple comparisons test for replicate experiments, with *n* = 4 for the control group and the *S*. *pneumoniae* + TIP peptide group and *n* = 5 for the *S*. *pneumoniae* + vehicle group as shown in **A** and **B**.

In a separate batch of donor lungs, AFC was measured at the start of perfusion and after 5 hours of perfusion to calculate the change in AFC over time and quantify alveolar epithelial dysfunction. Control lungs that did not receive bacteria maintained a relatively stable AFC over 5 hours of perfusion (Figure 3A), as AFC decreased modestly by 12% ± 8% (Figure 3B). Instillation of *S*. *pneumoniae* into the distal airspaces resulted in a marked decrease in AFC by 68% ± 15% after 3.5 hours compared with the AFC measured at baseline. In lungs that were administered the TIP peptide following instillation of *S*. *pneumoniae*, AFC decreased by 26% ± 18%, significantly less than in lungs that received only the bacteria without the TIP peptide (Figure 3B).

### Immune cell infiltration into the airspaces in response to S. pneumoniae is not altered by the TIP peptide.

**Figure 4:**
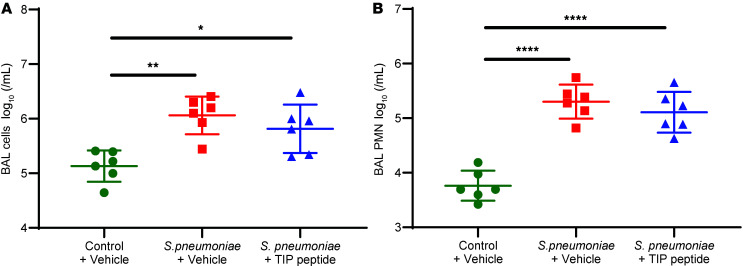
The TIP peptide does not alter the infiltration of immune cells in response to *S*. *pneumoniae* in the distal airspaces of the human lung. (**A**) The number of cells retrieved in the BAL from the distal airspaces was significantly greater in the human lungs that were instilled with *S*. *pneumoniae* than the control lungs that did not receive bacteria. Treatment with the TIP peptide resulted in a similarly significantly greater number of cells in the BAL than in control lungs that was comparable to the levels in the lungs that received bacteria but not the TIP peptide. (**B**) A sample of the BAL was used to determine the cell differential and measure the number of polymorphonuclear neutrophils (PMNs) in the BAL, which was significantly greater in the human lungs that were instilled with *S*. *pneumoniae* than in control lungs. The treatment with the TIP peptide did not change the number of PMNs in the BAL compared to lungs that received bacteria but not the TIP peptide. All data are presented as the base-10 log transformation of the number of cells or PMNs/mL. *P < 0.05, **P < 0.01, ****P < 0.0001, 1-way ANOVA with Tukey’s multiple comparisons test for replicate experiments, with *n* = 6 for each group shown in **A** and **B**.

The total number of cells (Figure 4A) and the number of neutrophils (Figure 4B) retrieved in the BAL were significantly higher from lungs that were instilled with *S*. *pneumoniae* compared with control lungs, indicating the bacteria in the distal airspaces had chemoattracted immune cells into the lung. Administering the TIP peptide following *S*. *pneumoniae* instillation did not significantly alter the number of cells or the number of neutrophils in the BAL, which remained higher than in the BAL obtained from control lungs.

### The TIP peptide does not impair bacterial clearance in the airspaces and reduces S. pneumoniae translocation into the circulation.

**Figure 5:**
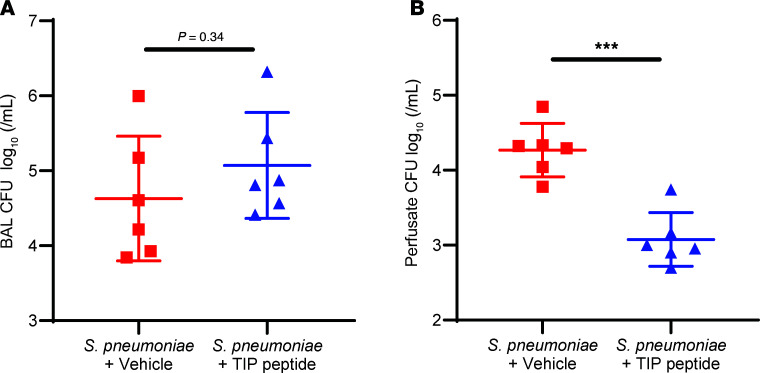
The TIP peptide does not impair bacterial clearance in the airspaces and reduces the translocation of *S*. *pneumoniae* into the circulation. (**A**) The number of CFU of *S*. *pneumoniae* retrieved in the BAL from the distal airspaces was not significantly different between human lungs that were treated with the TIP peptide and those untreated. In both experimental groups, the CFU in the BAL was substantially lower than in the initial inoculum (5 × 10^10^ CFU), indicating the lungs had cleared many of the bacteria from the airspaces during perfusion. (**B**) The number of CFU of *S*. *pneumoniae* measured in the circulating perfusate after 6 hours of perfusion was significantly lower in the human lungs treated with the TIP peptide compared with untreated lungs. All data are presented as the base-10 log transformation of the number of CFU/mL. ***P < 0.001, Mann-Whitney *U* test for replicate experiments, with *n* = 6 for each group shown in **A** and **B**.

At 4.5 hours after the instillation of *S*. *pneumoniae* into the distal airspaces, the number of bacteria retrieved in the BAL (Figure 5A) was 2 × 10^5^ CFU/mL on average, thus lower than the number of bacteria in the initial inoculum, 5 × 10^10^ CFU. The TIP peptide did not significantly change the number of CFU of *S*. *pneumoniae* in the BAL. However, the number of bacteria in the circulating perfusate at the end of perfusion was significantly lower in lungs treated with the TIP peptide compared with untreated lungs (Figure 5B).

### The levels of the pro-inflammatory cytokine IL-6 and the chemokine IL-8 in the airspaces are reduced by the TIP peptide.

**Figure 6:**
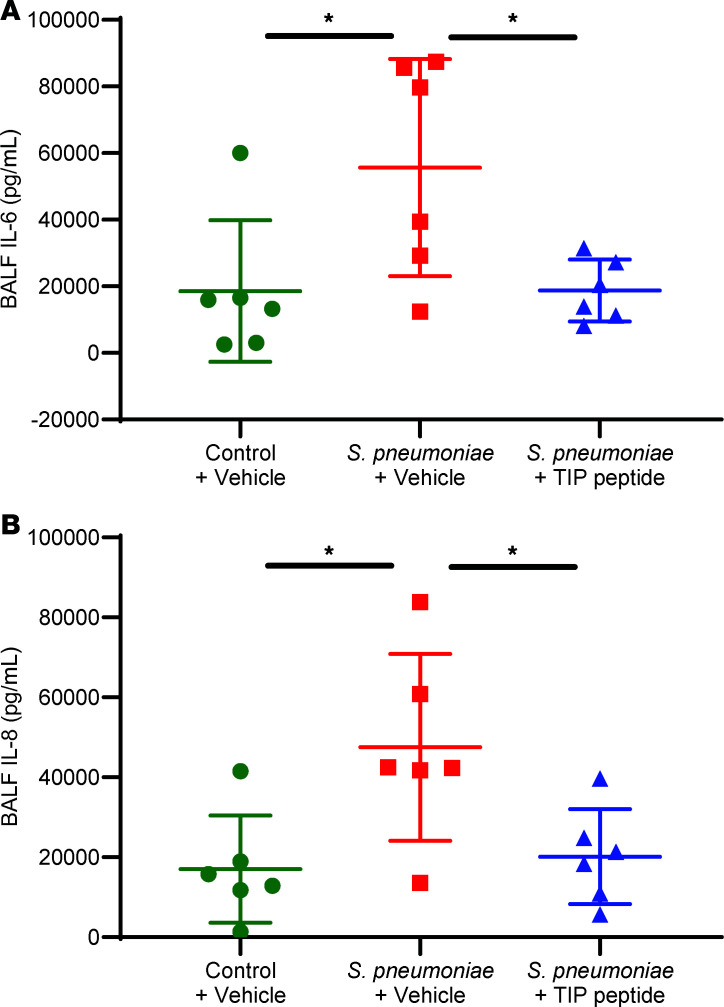
The TIP peptide reduces the levels of IL-6 and IL-8 in the airspaces. Concentrations of the cytokine IL-6 (**A**) and of the chemokine IL-8 (**B**) in the cell-free BALF were significantly increased by *S*. *pneumoniae* instilled into the airspaces of the ex vivo–perfused human lung. Therapeutic administration of the TIP peptide following *S*. *pneumoniae* instillation significantly reduced IL-6 and IL-8 concentrations in the BALF to levels comparable to control lungs that did not receive bacteria. *P < 0.05, 1-way ANOVA with Tukey’s multiple comparisons test for replicate experiments, with *n* = 6 for each group shown in **A** and **B**.

To assess the effect of bacteria and the TIP peptide on biochemical indices of inflammation, the concentrations of a panel of cytokines/chemokines and biomarkers were measured in the cell-free BALF. *S*. *pneumoniae* instilled into the distal airspaces significantly increased concentration of the pro-inflammatory cytokine IL-6 (Figure 6A) and the chemokine IL-8 (Figure 6B) in the BALF compared with control lungs that did not receive bacteria. These measurements were made to test for relevance to our clinical studies of biomarkers in patients with ARDS, in which we have determined that the hyperinflammatory phenotype, characterized by increased IL-6 and IL-8 generation, is associated with higher mortality (35). Treatment with the TIP peptide significantly reduced the BALF concentrations of IL-6 and IL-8 compared with injured lungs that were not treated with the TIP peptide, to levels comparable to those of control lungs (Figure 6). Concentrations of other biomarkers measured in the BALF were not significantly affected, as shown in Supplemental Figure 1; supplemental material available online with this article; https://doi.org/10.1172/jci.insight.188325DS1

## Discussion

The primary findings from this study establish that the lectin-like domain of TNF, mimicked by the TIP peptide, reduces lung endothelial and alveolar epithelial barrier permeability and enhances AFC in a translationally relevant model of acute bacterial pneumonia. The combined effect of the TIP peptide on these 2 mechanisms results in reduced pulmonary edema formation in the ex vivo–perfused human lung injured by *S*. *pneumoniae*. In addition, the TIP peptide also decreases the airspace concentration of the pro-inflammatory mediators IL-6 and IL-8 and reduces translocation of bacteria from the airspaces to the circulation.

Pneumonia is a major risk factor for developing ARDS, and *S*. *pneumoniae* is the primary pathogen causing bacterial pneumonia and lung injury (2). Although several candidate therapeutics have been studied in clinical trials, there is no proven pharmacological treatment for ARDS; lung-protective mechanical ventilation and prone positioning remain the only proven interventions. *S*. *pneumoniae* and PLY, its pore-forming toxin, disrupt the barrier integrity of capillary endothelial cells, leading to extravasation of protein-rich edema fluid into the lung interstitium and airspaces through increased permeability of the alveolar-capillary barrier (36). Thus, endothelial barrier disruption is a key factor in developing pulmonary edema, and targeting the endothelium may be critical for treating ARDS (37). Prior studies have reported that the TIP peptide preserved the barrier integrity of pulmonary endothelial cells injured by PLY in vitro and reduced PLY-induced capillary leak in vivo (38, 39). The TIP peptide also protected lung endothelial barrier integrity in an in vitro COVID-19 model using the S1 subunit of the SARS-CoV-2 spike protein to injure endothelial cells (40). These barrier-protective effects are mediated by activation of ENaC by the TIP peptide, which leads to inhibition of Ca^2+^-dependent mechanisms of barrier disruption induced by PLY (23). Specifically, ENaC activation leads to the inhibition of Ca^2+^ influx–dependent protein kinase Cα activity, which, in turn, reduces NADPH oxidase 2–mediated superoxide formation and endothelial barrier disruption caused by pneumococcal infection in mice (41). Moreover, ENaC activation by the TIP peptide also promotes the generation of nitric oxide, which has a protective effect on the endothelial barrier (42). In this study, weight gain (Figure 2A), a surrogate for edema formation, and BALF total protein concentration (Figure 2B), a measure of alveolar-capillary barrier permeability, were both decreased by TIP peptide treatment in perfused human lungs injured by *S*. *pneumoniae*. Thus, the TIP peptide reduced the endothelial barrier dysfunction triggered by *S*. *pneumoniae*.

The strengthening of the pulmonary endothelial barrier induced by the TIP peptide may explain the reduction in the number of CFU of *S*. *pneumoniae* detected in the perfusate (Figure 5B) at the end of perfusion. It is likely that the tighter barrier due to TIP peptide treatment reduced the degree of permeability through which the bacteria could translocate from the airspaces into the circulation. This reduction in bacterial translocation to the circulation suggests that the TIP peptide may reduce the risk of bacteremia and, thereby, decrease the potential for the infection spreading to other organs and the development of sepsis, an important complication of pneumococcal pneumonia (43).

In addition to disrupting the pulmonary barrier, PLY impairs the function and expression of ENaC in the alveolar epithelium and reduces AFC, which can contribute to the accumulation of edema fluid in the airspaces, a primary feature of ARDS (44). Accordingly, in this study, there was a significant decrease in AFC 3.5 hours after the instillation of *S*. *pneumoniae* into the distal airspaces of the perfused human lungs (Figure 3), an effect that was reversed by treatment with the TIP peptide. Prior studies have shown that the TIP peptide binds to the ENaC-α subunit and increases the current flowing through the channel in addition to preserving ENaC expression (15). Taken together, the combined effects of the TIP peptide improved AFC and reduced pulmonary edema in the human lung.

Pneumococcal infection in the lungs triggers the infiltration of PMNs into the airspaces as a first line of defense to control the infection and clear the bacteria (45). This was documented by the more than 10-fold greater number of PMNs detected in the airspaces of ex vivo–perfused human lungs instilled with *S*. *pneumoniae* compared with control lungs that were not instilled with bacteria (Figure 4). The PMNs detected in the BAL were primarily from the fresh whole blood that was added to the circulating perfusate and accumulated in the airspaces during the perfusion, as the donor lungs contain relatively few PMNs in the airspaces at baseline prior to perfusion (31). Alveolar macrophages (AMs) also participate in the clearance of bacteria and in repair, as others and we have reported in prior studies, including those using the ex vivo–perfused human lung preparation (31, 46). Accordingly, the number of *S*. *pneumoniae* remaining in the airspaces and retrieved in the BAL after 4.5 hours of perfusion was significantly lower than the 5 × 10^10^ CFU that was instilled in the initial inoculum (Figure 5A), demonstrating the human lungs perfused with whole blood had the capacity to clear some of the bacteria from the airspaces. Moreover, treatment with the TIP peptide did not impair chemotaxis of PMNs into the airspaces and did not reduce the clearance of bacteria from the airspaces, which are important preclinical safety findings.

The TIP peptide also significantly reduced IL-6 and IL-8 in the distal airspaces (Figure 6) to levels comparable to control lungs, suggesting an antiinflammatory effect of the TIP peptide. This effect has previously been reported in an in vivo murine model of nephrotoxic serum nephritis (42). BALF levels of other proteins that were measured in this panel of common biomarkers measured in ARDS (47) were not significantly changed by *S*. *pneumoniae* instillation or by TIP peptide treatment. The reduction of IL-6 and IL-8 in the BALF suggests that the TIP peptide might be particularly effective in the hyperinflammatory phenotype of ARDS, which is characterized by elevated levels of IL-6 and IL-8 (35). Our recent clinical study indicates that the levels of IL-6 and other biomarkers can be measured in real time in patients with ARDS (48). Thus, future ARDS clinical trials could test the TIP peptide specifically in patients with the hyperinflammatory phenotype. Additionally, levels of surfactant protein D (SP-D) in the BALF were elevated by the TIP peptide (Supplemental Figure 1G), suggesting that the TIP peptide might modulate metabolic processing of SP-D, which is produced by alveolar epithelial type 2 cells and is recycled by AMs (49). Our prior clinical studies have reported that lower levels of SP-D in the airspaces were associated with lung injury (50). Nonetheless, the increase in SP-D with TIP peptide treatment warrants further investigation in future studies.

There are some limitations to this study. The TIP peptide was instilled via the airspaces instead of the perfusate, but this route of administration is likely optimal as clinical trials are administering the TIP peptide via inhalation (51), and it targets the alveolar epithelium as well as the lung endothelium. Also, the experiments were conducted in the ex vivo–perfused human lung over only 6 hours by necessity, but the findings do provide translational evidence for the protective effects of the TIP peptide in a human lung model of bacterial pneumonia and add to the growing evidence supporting studies of the TIP peptide (also known as solnatide and AP-301) in clinical trials of ARDS (23). A phase I double-blind, placebo-controlled trial found no adverse effects associated with a single dose of inhaled TIP peptide (up to 120 mg) in 48 healthy male individuals (52). Subsequently, a phase IIa double-blind, placebo-controlled trial of 40 mechanically ventilated patients with ARDS showed that inhaled TIP peptide significantly reduced ventilator pressures and the extravascular lung water index over 7 days in patients with an initial sequential organ failure assessment score of at least 11 compared with placebo (53). Additionally, a randomized, placebo-controlled pilot study of 20 lung transplant recipients with primary graft dysfunction demonstrated that inhaled TIP peptide improved gas exchange and shortened the duration of mechanical ventilation over 7 days compared with placebo (54). There is currently an ongoing dose-escalating, multicenter, phase IIb, randomized, placebo-controlled, double-blind trial of inhaled TIP peptide in patients with moderate to severe ARDS (51). The promising results from this study in the ex vivo–perfused human lung may warrant a clinical trial that tests the TIP peptide specifically in ARDS patients with bacterial pneumonia. Trials of different therapeutics for ARDS in the past may have failed because they did not sufficiently target the potentially optimal treatment-responsive ARDS patient group (4, 55).

In conclusion, *S*. *pneumoniae* instilled into the distal airspaces of the ex vivo–perfused human lung caused pulmonary edema from injury to the lung endothelial and epithelial barriers and by impairing AFC. Therapeutic administration of the TIP peptide reduced the magnitude of pulmonary edema by decreasing lung endothelial injury and attenuating the reduction in AFC. The TIP peptide also reduced translocation of bacteria into the circulation and had an antiinflammatory effect in the airspaces. Thus, the findings from these experiments provide translational evidence for the TIP peptide as a promising potential therapy for critically ill patients with pneumococcal pneumonia.

## Methods

Further information can be found in Supplemental Methods.

### Sex as a biological variable.

This study used human lungs procured from both male and female donors, and similar findings are reported for both sexes.

### Ex vivo human lung perfusion preparation.

Donor human lungs not used for transplant were procured for research purposes by Donor Network West and immediately transported in 4°C to the Matthay lab at UCSF. Either the left or the right lung was selected for perfusion based on the absence of injury by gross appearance. The PA was cannulated by purse string suture, and a 7.5 mm endobronchial tube (ET) was secured by suturing into the main bronchus of the lung. The lung was then placed in a humidified plexiglass chamber containing a reservoir at the bottom with 2 L of perfusate, which was a mixture of 5% bovine serum albumin (GeminiBio) in DMEM, high glucose (DME-H21, UCSF Media Production Core Facilities). The perfusate was maintained at 37°C by a water bath. The PA canula was connected to the roller pump (Sarns MDX 7000) circuit, and the perfusate was circulated at a rate of 0.2 L/min for a PA pressure of 10 mmHg. Once the perfusate freely draining from the pulmonary vein reached 37°C, the lung was inflated with 8 cmH_2_O of CPAP to begin the experiment.

### Measurement of AFC.

Once the lung was inflated by CPAP, a second ET (3 mm) was inserted through a side port of the first ET to reach the distal airways of the lower lobe. A total of 100 mL of 0.9% saline solution containing 5% bovine serum albumin (GeminiBio) warmed to 37°C was instilled through the second ET into the distal airspaces. Five minutes afterward, a catheter (PE-240 tubing, Becton-Dickinson) was inserted through the second ET to reach the distal airspaces, and a small volume (~200 μL) of alveolar fluid was sampled; a second sample of alveolar fluid was obtained after 30 minutes. A refractometer (Atago) was used to measure the total protein concentration in the 2 alveolar fluid samples (collected at 5 and 35 minutes) to calculate AFC according to the formula: AFC (%/h) = 2(1 – C_i_/C_f_), where C_i_ and C_f_ are the protein concentrations in the 5-minute and 35-minute samples, respectively, as done in our prior studies (30, 31). AFC was measured again in the same region of the lower lobe after 5 hours of perfusion to calculate a percentage change in AFC between 0 hour and 5 hours. Supplemental Table 1 lists the relevant characteristics of the donors for the lungs in which AFC measurement studies were performed, including the donor age, the cold-ischemia time for the lungs, and the last ratio of measured arterial partial pressure of O_2_ and fraction of inspired O_2_ (PaO_2_/FiO_2_) for the donor, which were not significantly different between the experimental groups. Following the initial AFC measurement, 100 mL of fresh whole blood was collected from a healthy volunteer in heparinized blood collection tubes and then added to the circulating perfusate through a side port of the PA cannula.

### Bacterial culture and intrabronchial instillation.

*S*. *pneumoniae* serotype 19F (ATCC) was cultured in autoclaved brain-heart broth (Becton-Dickinson) to mid-log phase (optical density 0.50 at 600 nm) in a 37°C incubator. The bacteria were then harvested by centrifugation (3,000*g*, 10 minutes, 4°C). For consistency across experiments, all bacterial cultures were derived from aliquots of a single bacterial expansion that were stored at –80°C. A total of 5 × 10^10^ CFU of bacteria were resuspended in 10 mL of 0.9% saline and then instilled through the second ET (3 mm) wedged in the distal airspaces of the lower lobe; a second volume of 5 mL of saline was subsequently instilled to flush the tube. Lungs in the control experimental group did not receive bacteria, and instead equivalent volumes of only saline vehicle were instilled. For experiments in which AFC was measured, the bacteria or vehicle was instilled into the same region of the lower lobe where AFC measurements were performed.

### Administration of the TIP peptide.

TIP peptide (also known as solnatide and AP-301) is a 17–amino acid cyclic synthetic peptide with the sequence CGQRETPEGAEAKPWYC and was produced by BCN Peptides and provided by Apeptico GmbH. The lyophilized TIP peptide was resuspended in 0.9% saline at a concentration of 10 mg/mL and stored in aliquots at –80°C. For each experiment, a frozen aliquot of 3.5 mg of TIP peptide was thawed and immediately diluted in 10 mL of 0.9% saline, then instilled through the second ET (3 mm) wedged into the distal airspaces in the same region of the lower lobe where bacteria were previously instilled; a second volume of 5 mL of saline was subsequently instilled to flush the tube. In lungs that did not receive the TIP peptide, equivalent volumes of only saline vehicle were instilled.

### Pulmonary edema measurements.

In a separate set of experiments using human lungs from a different group of donors, AFC was not measured, but the same procedures described above for bacterial instillation and TIP peptide administration were performed. Supplemental Table 2 lists the key characteristics of the donors for these lungs, including the donor age, the cold-ischemia time for the lungs, and the last measured PaO_2_/FiO_2_ ratio, which were not significantly different between the experimental groups. In these experiments, the weight of the lung was measured prior to perfusion and again after 6 hours of perfusion. The resulting initial weight (w_i_) and final weight (w_f_) of the lung were used to calculate a percentage weight gain according to the formula: % weight gain = (w_f_/w_i_ – 1) × 100%. The percentage weight gain was used as an index of pulmonary edema formation during perfusion.

### BAL and cell counting.

After measuring the final weight of the lung at 6 hours of perfusion, BAL was performed by instilling 100 mL of 0.9% saline through the second ET (3 mm) wedged into the distal airspaces of the lower lobe in the same region where bacteria or vehicle and the TIP peptide or vehicle were previously instilled. The BAL that was subsequently retrieved from the lung was filtered through a 100 μm pore sieve (Corning) to remove debris. The number of cells in the filtered BAL were counted using an automated cell counter (Cytosmart, Corning). A sample of BAL was cytocentrifuged onto a microscope slide and then stained with the Hema 3 system (Thermo Fisher Scientific). Stained cells were visualized under a microscope (Nikon), and the cell differential was determined from counting a total of 100 cells for each sample. The remaining BAL sample was centrifuged (1,000*g*, 10 minutes, 4°C) to pellet the cells, and the resulting supernatant BALF was stored at –80°C.

### Counting CFU of bacteria.

At the end of 6 hours of perfusion, a sample of perfusate was collected from the perfusion circuit. The perfusate sample was serially diluted, and each dilution and the undiluted sample were seeded onto sheep blood agar plates, which were then placed in a 37°C incubator overnight. The number of CFU of *S*. *pneumoniae* were counted in plates with a sample dilution that produced a reliably countable number of colonies. The BAL sample was also similarly serially diluted and seeded onto sheep blood agar plates to count the number of CFU recovered from the airspaces.

### Measurement of total protein and biomarkers.

Total protein concentration in the cell-free BALF was measured using the Pierce bicinchoninic acid assay (Thermo Fisher Scientific) and a Synergy plate reader (BioTek, Agilent). Concentrations of cytokines and biomarkers in the BALF were measured with the Luminex multiplex platform using the Human Luminex Discovery Assay kits for the targeted analytes (R&D Systems, Bio-Techne), and the quantified analytes were measured on the FLEXMAP instrument with raw data analyzed using the xPONENT software.

### Statistics.

All data were analyzed using Prism 10 software (GraphPad, Inc.) and are presented as mean and standard deviation, with each data point representing 1 replicate. A *P* < 0.05 was used to determine statistical significance between experimental groups using 1-way ANOVA with Tukey’s multiple comparisons test or the Mann-Whitney *U* test.

### Study approval.

These studies were done using lungs from cadaver donors. They do not require UCSF human research institutional review board approval. The donation of organs for research was approved by the family of the deceased donor through Donor Network West, the organ procurement organization, San Ramon, California, USA.

### Data availability.

Values for individual data points in figures and tables are reported in the Supporting Data Values file.

## Author contributions

MM, BE, HT, and SM performed the ex vivo human lung perfusion experiments, functional measurements, and sample collections. AR and CL performed cell, protein, and biomarker measurements. MM, RL, and MAM contributed to study design and interpretation of data. MM prepared and revised the manuscript, and RL and MAM reviewed and edited the manuscript. All authors reviewed and approved the final version of the manuscript before submission.

## Supplementary Material

Supplemental data

Supporting data values

## Version 1 06/09/2025

Electronic publication

## References

[1] Matthay MA (2019). Acute respiratory distress syndrome. Nat Rev Dis Primers.

[2] Cilloniz C (2018). Acute respiratory distress syndrome in mechanically ventilated patients with community-acquired pneumonia. Eur Respir J.

[3] Bos LDJ, Ware LB (2022). Acute respiratory distress syndrome: causes, pathophysiology, and phenotypes. Lancet.

[4] Wick KD (2021). Promises and challenges of personalized medicine to guide ARDS therapy. Crit Care.

[5] Herrero R (2018). New insights into the mechanisms of pulmonary edema in acute lung injury. Ann Transl Med.

[6] Ware LB, Matthay MA (2001). Alveolar fluid clearance is impaired in the majority of patients with acute lung injury and the acute respiratory distress syndrome. Am J Respir Crit Care Med.

[7] Huppert LA, Matthay MA (2017). Alveolar fluid clearance in pathologically relevant conditions: *in vitro* and *in vivo* models of acute respiratory distress syndrome. Front Immunol.

[8] Verkman AS (2000). Aquaporin water channels and lung physiology. Am J Physiol Lung Cell Mol Physiol.

[9] Eaton DC (2009). The contribution of epithelial sodium channels to alveolar function in health and disease. Annu Rev Physiol.

[10] Hummler E (1996). Early death due to defective neonatal lung liquid clearance in alpha-ENaC-deficient mice. Nat Genet.

[11] Lemmens-Gruber R, Tzotzos S (2023). The epithelial sodium channel-an underestimated drug target. Int J Mol Sci.

[12] Lucas R (2012). Agonist of growth hormone-releasing hormone reduces pneumolysin-induced pulmonary permeability edema. Proc Natl Acad Sci U S A.

[13] Migneault F (2013). Cycloheximide and lipopolysaccharide downregulate αENaC mRNA via different mechanisms in alveolar epithelial cells. Am J Physiol Lung Cell Mol Physiol.

[14] Lucas R (2016). The lectin-like domain of TNF increases ENaC open probability through a novel site at the interface between the second transmembrane and C-terminal domains of the α-subunit. J Biol Chem.

[15] Czikora I (2014). A novel tumor necrosis factor-mediated mechanism of direct epithelial sodium channel activation. Am J Respir Crit Care Med.

[16] Yamagata T (2009). The regulation of amiloride-sensitive epithelial sodium channels by tumor necrosis factor-alpha in injured lungs and alveolar type II cells. Respir Physiol Neurobiol.

[17] Fukuda N (2001). Mechanisms of TNF-alpha stimulation of amiloride-sensitive sodium transport across alveolar epithelium. Am J Physiol Lung Cell Mol Physiol.

[18] Rezaiguia S (1997). Acute bacterial pneumonia in rats increases alveolar epithelial fluid clearance by a tumor necrosis factor-alpha-dependent mechanism. J Clin Invest.

[19] Börjesson A (2000). TNF-alpha stimulates alveolar liquid clearance during intestinal ischemia-reperfusion in rats. Am J Physiol Lung Cell Mol Physiol.

[20] Tillie-Leblond I (2002). Chronic bronchial allergic inflammation increases alveolar liquid clearance by TNF-alpha -dependent mechanism. Am J Physiol Lung Cell Mol Physiol.

[21] Hamacher J (2017). Cytokine-ion channel interactions in pulmonary inflammation. Front Immunol.

[22] Wilson MR (2007). Differential roles of p55 and p75 tumor necrosis factor receptors on stretch-induced pulmonary edema in mice. Am J Physiol Lung Cell Mol Physiol.

[23] Lucas R (2021). Dichotomous role of tumor necrosis factor in pulmonary barrier function and alveolar fluid clearance. Front Physiol.

[24] Lucas R (1994). Mapping the lectin-like activity of tumor necrosis factor. Science.

[25] Tzotzos S (2013). AP301, a synthetic peptide mimicking the lectin-like domain of TNF, enhances amiloride-sensitive Na(+) current in primary dog, pig and rat alveolar type II cells. Pulm Pharmacol Ther.

[26] Shabbir W (2013). Mechanism of action of novel lung edema therapeutic AP301 by activation of the epithelial sodium channel. Mol Pharmacol.

[27] Elia N (2003). Functional identification of the alveolar edema reabsorption activity of murine tumor necrosis factor-alpha. Am J Respir Crit Care Med.

[28] Hamacher J (2010). The lectin-like domain of tumor necrosis factor improves lung function after rat lung transplantation--potential role for a reduction in reactive oxygen species generation. Crit Care Med.

[29] Lucas R (2011). The TNF-derived TIP peptide reduces lung dysfunction in experimental influenza A virus infection. Eur Respir J.

[30] Ross JT (2019). The ex vivo human lung: research value for translational science. JCI Insight.

[31] Ross JT (2020). The ex vivo perfused human lung is resistant to injury by high-dose *S*. *pneumoniae* bacteremia. Am J Physiol Lung Cell Mol Physiol.

[32] Gennai S (2015). Microvesicles derived from human mesenchymal stem cells restore alveolar fluid clearance in human lungs rejected for transplantation. Am J Transplant.

[33] Lee JW (2009). Allogeneic human mesenchymal stem cells for treatment of E. coli endotoxin-induced acute lung injury in the ex vivo perfused human lung. Proc Natl Acad Sci U S A.

[34] Lee JW (2013). Therapeutic effects of human mesenchymal stem cells in ex vivo human lungs injured with live bacteria. Am J Respir Crit Care Med.

[35] Sinha P (2020). Development and validation of parsimonious algorithms to classify acute respiratory distress syndrome phenotypes: a secondary analysis of randomised controlled trials. Lancet Respir Med.

[36] Nishimoto AT (2020). Pneumolysin: pathogenesis and therapeutic target. Front Microbiol.

[37] Millar FR (2016). The pulmonary endothelium in acute respiratory distress syndrome: insights and therapeutic opportunities. Thorax.

[38] Lucas R (2012). Protein kinase C-α and arginase I mediate pneumolysin-induced pulmonary endothelial hyperpermeability. Am J Respir Cell Mol Biol.

[39] Czikora I (2017). Epithelial sodium channel-α mediates the protective effect of the TNF-derived TIP Peptide in pneumolysin-induced endothelial barrier dysfunction. Front Immunol.

[40] Romero MJ (2023). Direct endothelial ENaC activation mitigates vasculopathy induced by SARS-CoV2 spike protein. Front Immunol.

[41] Romero MJ (2024). Endothelial ENaC-α restrains oxidative stress in lung capillaries in murine pneumococcal pneumonia-associated acute lung injury. Am J Respir Cell Mol Biol.

[42] Madaio MP (2019). The TNF-derived TIP peptide activates the epithelial sodium channel and ameliorates experimental nephrotoxic serum nephritis. Kidney Int.

[43] Kruckow KL (2023). Acute organ injury and long-term sequelae of severe pneumococcal infections. Pneumonia (Nathan).

[44] Lucas R (2013). Mini-review: novel therapeutic strategies to blunt actions of pneumolysin in the lungs. Toxins (Basel).

[45] Grudzinska FS (2020). Neutrophils in community-acquired pneumonia: parallels in dysfunction at the extremes of age. Thorax.

[46] Arafa EI (2022). Recruitment and training of alveolar macrophages after pneumococcal pneumonia. JCI Insight.

[47] Matthay MA (2020). Phenotypes and personalized medicine in the acute respiratory distress syndrome. Intensive Care Med.

[48] https://www.atsjournals.org/doi/abs/10.1164/ajrccm-conference.2024.209.1_MeetingAbstracts.A5512.

[49] Sorensen GL (2018). Surfactant protein D in respiratory and non-respiratory diseases. Front Med (Lausanne).

[50] Cheng IW (2003). Prognostic value of surfactant proteins A and D in patients with acute lung injury. Crit Care Med.

[51] Schmid B (2021). Safety and preliminary efficacy of sequential multiple ascending doses of solnatide to treat pulmonary permeability edema in patients with moderate-to-severe ARDS-a randomized, placebo-controlled, double-blind trial. Trials.

[52] Schwameis R (2014). A FIM study to assess safety and exposure of inhaled single doses of AP301-A specific ENaC channel activator for the treatment of acute lung injury. J Clin Pharmacol.

[53] Krenn K (2017). Inhaled AP301 for treatment of pulmonary edema in mechanically ventilated patients with acute respiratory distress syndrome: a phase IIa randomized placebo-controlled trial. Crit Care.

[54] Aigner C (2018). Treatment of primary graft dysfunction after lung transplantation with orally inhaled AP301: a prospective, randomized pilot study. J Heart Lung Transplant.

[55] Calfee CS (2018). Acute respiratory distress syndrome subphenotypes and differential response to simvastatin: secondary analysis of a randomised controlled trial. Lancet Respir Med.

