# Supplementary material for: The lectin-like domain of TNF reduces pneumonia-induced injury in the perfused human lung
Source: JCI Insight. 2025 Jun 9;10(11):e188325. doi: 10.1172/jci.insight.188325 (PMC12226048; doi:10.1172/jci.insight.188325)
Supplement: Supplemental data [file jciinsight-10-188325-s019.pdf]

Supplementary Material for

The lectin-like domain of tumor necrosis factor reduces pneumonia-induced injury in the perfused human lung

Mazharul Maishan[1], Hiroki Taenaka[1], Bruno Evrard[2,3], Shotaro Matsumoto[1], Angelika Ringor[1], Carolyn Leroux[1], Rudolf Lucas[4,5,6*], and Michael A. Matthay[1,7,8*]

[1]Cardiovascular Research Institute, University of California, San Francisco, CA, USA, [2]Division of Pulmonary, Critical Care, Allergy and Sleep Medicine, Department of Medicine, University of California, San Francisco, CA, USA, [3]Inserm CIC 1435, Dupuytren Teaching Hospital, Limoges, France, [4]Vascular Biology Center, [5]Department of Pharmacology and Toxicology and [6]Division of Pulmonary Critical Care and Sleep Medicine, Medical College of Georgia, Augusta University, Augusta, GA, USA, [7]Department of Medicine, University of California, San Francisco, CA, USA, [8]Department of Anesthesiology, University of California, San Francisco, CA, USA

Corresponding author: Mazharul Maishan, University of California, San Francisco, 513 Parnassus Ave, HSE760, San Francisco, California 94143, USA. Phone: +1 415.476.1079; E-mail: mazharul.maishan@ucsf.edu.

***Co-senior authors:** Rudolf Lucas and Michael A. Matthay

Methods

Donor human lungs. Alveolar fluid clearance measurements were performed in one batch of human lungs that were procured for research by Donor Network West and the relevant donor characteristics for these lungs are listed in **Supplementary Table 1**. A separate batch of human lungs from a different group of donors were procured for studies measuring weight gain and for bronchoalveolar lavage, and the relevant donor characteristics for these lungs are listed in **Supplementary Table 2**.

Measurement of biomarkers. Concentrations of cytokines and biomarkers in the cell-free bronchoalveolar lavage fluid (BALF) were measured with the Luminex multiplex platform using the Human Luminex Discovery Assay kits for the targeted analytes (R&D Systems) and the quantified analytes were measured on the FLEXMAP instrument with raw data analyzed using the xPONENT software.

Statistical analysis. All data were analyzed using Prism 10 software (GraphPad, Inc.) and are presented as mean and standard deviation, with each data point representing one biological replicate. A P value < 0.05 was used to determine statistical significance between experimental groups using one-way ANOVA with Tukey's multiple comparisons test.

Data availability. Values for individual data points in figures and tables are reported in the Supporting Data Values file.

Figures

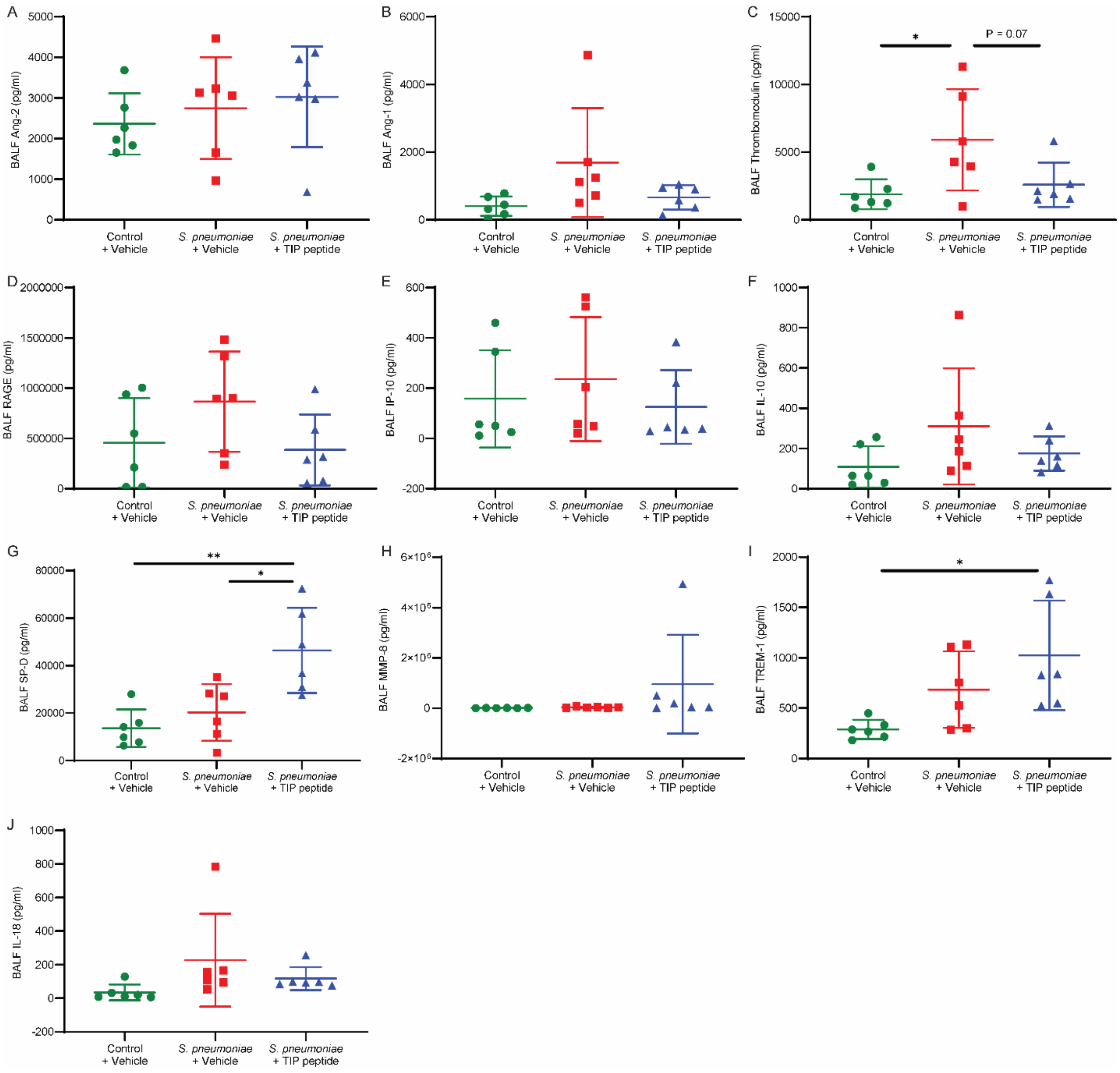

Supplementary Figure 1. Concentrations of biomarkers measured in the bronchoalveolar lavage fluid from the *ex vivo* perfused human lung. A panel of cytokines, chemokines and biomarkers measured in the cell-free bronchoalveolar lavage fluid (BALF) retrieved from the *ex vivo* perfused human lungs after 6 hours of perfusion. *P < 0.05, **P < 0.01, one-way ANOVA with Tukey’s multiple comparisons test for replicate experiments, with n=6 for each group shown in panels A and B.

Tables

	Control + Vehicle (n = 4)	SP + Vehicle (n = 5)	SP + TIP peptide (n = 4)
Sex	1 Male / 3 Female	2 Male / 3 Female	1 Male / 3 Female
Age (years)	48 ± 22	45 ± 12	45 ± 17
DCD donor (Yes/No)	0/4	1/4	0/4
Cold-ischemia time (hours)	31 ± 9	26 ± 11	30 ± 9
Last PaO2/FiO2 ratio	381 ± 79	357 ± 91	375 ± 191

Supplementary Table 1. Relevant donor characteristics for the human lungs used in *ex vivo* perfusion studies for measurements of alveolar fluid clearance. There were no significant differences between the experimental groups in the age of the donor, the cold-ischemia time of the lungs, or the last measured PaO2/FiO2 ratio for the donor. Out of a total of 13 donors, 1 was a donor after circulatory death (DCD). SP = *Streptococcus pneumoniae*.

	Control + Vehicle (n = 6)	SP + Vehicle (n = 6)	SP + TIP peptide (n = 6)
Sex	4 Male / 2 Female	5 Male / 1 Female	5 Male / 1 Female
Age (years)	47 ± 12	42 ± 9	39 ± 16
DCD donor (Yes/No)	2/4	0/6	0/6
Cold-ischemia time (hours)	25 ± 10	24 ± 10	30 ± 12
Last PaO2/FiO2 ratio	322 ± 117	296 ± 91	271 ± 107

Supplementary Table 2. Relevant donor characteristics for the human lungs used in *ex vivo* perfusion studies for measurements of weight gain and for bronchoalveolar lavage. There were no significant differences between the experimental groups in the age of the donor, the cold-ischemia time of the lungs, or the last measured PaO2/FiO2 ratio for the donor. Out of a total of 18 donors, 2 were donors after circulatory death (DCD) and both were in the control group. SP = *Streptococcus pneumoniae*.
